# A Brain-Penetrating Hsp90 Inhibitor NXD30001 Inhibits Glioblastoma as a Monotherapy or in Combination With Radiation

**DOI:** 10.3389/fphar.2020.00974

**Published:** 2020-06-30

**Authors:** Hao Chen, Yuanying Gong, Yufang Ma, Reid C. Thompson, Jialiang Wang, Zhixiang Cheng, Lixia Xue

**Affiliations:** ^1^ Department of Neurosurgery, Shanghai Jiao Tong University Affiliated Sixth People’s Hospital, Shanghai, China; ^2^ Department of Neurological Surgery, Vanderbilt University Medical Center, Nashville, TN, United States; ^3^ Department of Pain Management, Sir Run Run Hospital, Nanjing Medical University, Nanjing, China; ^4^ Department of Pain Management, Second Affiliated Hospital, Nanjing Medical University, Nanjing, China; ^5^ Department of Neurology, Shanghai Jiao Tong University Affiliated Sixth People’s Hospital, Shanghai, China

**Keywords:** glioblastoma, radiation resistance, Hsp90 inhibitor, molecular pharmacology, DNA damage response, ER stress

## Abstract

Glioblastoma multiforme (GBM) is a highly heterogeneous disease, which is initiated and sustained by various molecular alterations in an array of signal transduction pathways. Heat-shock protein 90 (Hsp90) is a molecular chaperone and is critically implicated in folding and activation of a diverse group of client proteins, many of which are key regulators for glioblastoma biology. We here assessed the anti-neoplastic efficacy of a novel brain-penetrating Hsp90 inhibitor NXD30001 as a monotherapy and combined with radiation *in vitro* and *in vivo*. Our results demonstrated that NXD30001 potently inhibited neurosphere formation, growth, and survival of CD133+ GBM cells with the half maximal inhibitory concentration at low nanomolar range, but CD133− GBM cells were less sensitive to NXD30001. NXD30001 also increased radio-sensitivity in glioblastoma stem cells (GSCs) at suboptimal concentrations. Moreover, NXD30001 dose-dependently decreased phosphorylation levels of multiple Hsp90 client proteins which play key roles in GBM, such as EGFR, Akt, c-Myc, and Notch1. In addition, NXD30001 could impair DNA damage response and endoplasmic reticulum stress response after radiotherapy by alteration of the related proteins expression. In a murine orthotopic model of human glioblastoma, NXD30001 marvelously induced tumor regression and extended median survival of tumor-bearing mice by approximately 20% when compared with the vehicle group (37 d vs 31 d, *P*<0.05). Radiotherapy solely increased median survival of tumor-bearing mice from 31 d to 38 d (*P*<0.05), while NXD30001 combined with radiation further extended survival to 43 d (*P*<0.05). We concluded that GSCs are more sensitive to NXD30001 than non-stem GBM cells, and NXD30001 in combination with radiation exerts better inhibitive effect in GBM progression than monotherapy.

## Introduction

Glioblastoma multiforme (GBM) is one of the most common malignant form of gliomas that with a median survival period of 12–15 months (Gittleman et al., 2018). Radiation therapy, the clinical application of ionizing radiation, is one crucial treatment option in modern GBM therapy apart from surgical resection and the chemotherapeutic alkylating agent (Stupp et al., 2005; Hathout and Pope, 2016). However, GBM is well documented for its rapid recurrence following radiotherapy, due to inadequate killing of cancer stem cells, which is known as tumor-initiating cells or tumor-propagating cells. CD133 is a pentaspan transmembrane protein and was commonly used as a glioblastoma stem cells (GSCs) biomarker, it has been showed that CD133 positive (CD133+) glioblastoma cells contributes to glioma radioresistance and tumor progress through preferential checkpoint response and DNA repair (Beier et al., 2007; Norollahi et al., 2019). *In vivo* and *in vitro* studies have also verified the fraction of CD133+ glioma cells were significantly increased after radiation treatment (Ahmed et al., 2018). Therefore, compromising the resistant mechanisms in these cells may significantly improve the efficacy of radiotherapy for GBM (Sheehan et al., 2010). However, the mechanisms implicated in GSCs radiation resistance remain poorly defined, which may involve combinatorial alterations in signaling networks that regulate DNA damage checkpoints, DNA repair, cellular survival, etc. (Skvortsova et al., 2015). As GBM is a highly heterogeneous disease, the radio-resistant mechanisms of GSCs drastically varied among tumors with distinguished molecular background. Therefore, the efficacy of single-targeted radio-sensitizing approaches is likely to be constrained to a small subset of patients.

The 90-kDa heat-shock protein (Hsp90) is a highly abundant molecular chaperone that is responsible for the maintenance of protein homeostasis under basal conditions and during stress response (Den and Lu, 2012). Hsp90 client proteins regulate a large number of cellular functions, including signal transduction, protein trafficking, chromatin remodeling, autophagy, cell proliferation, and survival (Zuehlke and Johnson, 2010). Many of these client proteins are frequently abnormally expressed in cancer cells and therefore inhibition of Hsp90 may be a rational approach to target cancer cells. Currently, several Hsp90 inhibitors have been examined in preclinical and clinical settings for different human cancers (Sidera and Patsavoudi, 2014). The Hsp90 inhibitors simultaneously target multiple radio-resistant pathways and thereby have preferential effects for GBM therapy (Camphausen and Tofilon, 2007). 17-Allylamino-17-demethoxygeldanamycin (17-AAG), a benzoquinone antibiotic derived from geldanamycin, is an Hsp90 inhibitor that has been shown to inhibit tumor growth in GBM either as a single agent or in combination with radiation (Sauvageot et al., 2009). However, 17-AAG and most other Hsp90 inhibitors cannot cross the blood-brain barrier (BBB) effectively, which greatly limited their potential efficacy for gliomas treatment. NXD30001, a novel radicicol-based series of Hsp90 inhibitors, has a more favorable brain pharmacokinetic profile and has been reported to inhibits Hsp90 potently than 17-AAG (Cha et al., 2014). NXD30001 could easily crosses the BBB and accumulates in the brain and would not cause liver or ocular toxicity *in vivo*, made it be an attractive therapeutic candidate for gliomas by downregulating Hsp90 client oncoproteins to inhibit tumor cell proliferation and induce cell apoptosis. Furthermore, previous studies depicted that NXD30001 could induce tumor regression in GBM model (Zhu et al., 2010) and neurofibromatosis type 2 model (Tanaka et al., 2013), which provides a compelling rationale for its use as an attractive therapeutic candidate in treatment for central nervous system tumors.

However, targeting Hsp90 solely faces several challenges. It has been reported that the anticancer efficacy of Hsp90 inhibitors as monotherapy in clinical trials was often less significant than predicted by preclinical models (Messaoudi et al., 2011). For example, Hsp90 may not be adequately inhibited in GBM or can be compensated by induction of co-chaperons, such as Hsp27 and Hsp70 (van Ommeren et al., 2016). Also, tumors *in vivo* may be less dependent on Hsp90 than *in vitro* (Neckers and Workman, 2012). As a preliminary study demonstrated that Hsp90 inhibitor could enhance the radiotherapy in a variety of human cancer cell lines, including GBM (Piper and Millson, 2011), we here explored the anti-neoplastic efficacy and mechanisms of NXD30001 as a monotherapy or in combination with radiation in GSCs and GBM orthotopic animal model.

## Materials and Methods

### Cell Culture and Enrichment of GSCs

The primary glioblastoma cell lines of T4105, T4302, and T4597 were generously provided by Dr. Jeremy Rich at Cleveland Clinic (Cleveland, OH). These tumor samples were originally derived from patient surgical specimens and serially passaged as subcutaneous xenograft tumors. Matched cultures enriched or depleted for the CD133+ glioma cells subpopulation was prepared following methods described in our previous publications (Wang et al., 2010b; Cheng et al., 2013; Ma et al., 2015; Gong et al., 2016; Ma et al., 2017). Briefly, cells were enzymatically dissociated from subcutaneous xenograft tumors and red blood cells were lysed in diluted phosphate-buffered saline solution (0.25×). The CD133+ and CD133 negative (CD133−) fractions were magnetically sorted using the CD133 Microbead kit (Miltenyi Biotec, Bergisch Gladbach, German) following the manufacturer’s instructions. Dissociated CD133+ cells or unsorted neurospheres were then cultured overnight in stem cell media (neurobasal media supplemented with B27, epidermal growth factor, and basic fibroblast growth factor at 20 ng/ml) before cell sorting for recovery of cellular surface antigens. CD133− cells were maintained in Dulbecco’s modified Eagle’s medium supplemented with 10% fetal bovine serum (Invitrogen, Carlsbad, CA) but were cultured in stem cell media at least 24 h prior to experiments to control differences in cell media. All cells were cultured at 37 °C with 5% CO_2_ and maintained for no more than five passages as these cells may spontaneously differentiate *in vitro*.

### Irradiation

Single cell suspension or monolayer culture for CD133+ cells were irradiated in medium with Varian 600 CD X-ray linear accelerator (Varian Medical Systems, Inc., Palo Alto, CA, USA) at a dose rate of 1, 2, or 3 Gy/min exposed at room temperature. Tumor-bearing mice receive whole brain X-ray radiation for 6 consecutive days at 3 Gy/day under anesthesia, and then tumors were measured by bioluminescence images.

### Reagent and Antibodies

NXD30001 was obtained from NexGenix Pharmaceuticals, 17AAG was generously provided by Kosan Bioscience, Inc. The antibodies used in this study including Phospho(p)-EGFR (1H12, #2236), EGFR (D6B6, #2085), pS473-Akt (D9E, #4060), Akt (40D4, #2920), Cleaved-Notch1 (D3B8, #4147), Notch1 (D1E11, #3608), c-Myc (D84C12, #5605), p-MEK (Thr286, #9127), p-ERK1/2 (D13.14.4E, #4370), ERK1/2 (L34F12, #4696), p-ATR (Ser428, #2853), p-CHK2 (Thr68, #2661), p-p53 (Ser15, #9286), p-H2AX (D7T2V, #80312), PERK (D11A8, #5683), IRE1α (14C10, #3294), BiP (C50B12, #3177), and CHOP (L63F7, #2895) were purchased from Cell signaling Technology. Mouse monoclonal antibody against actin (#MAB1501) was purchased from Millipore. Secondary antibodies were obtained from Santa Cruz Biotechnology, Inc.

### Cell Viability, Neurosphere Formation Assay, and Apoptosis Detection

GSC-enriched spheroid cultures (spheres) were used to test cell viability and neurospheres formation. For cell viability assay, cells were seeded into white, 96-Well White Flat Bottom (Corning, NY, USA) at 5,000 cells per well in a total volume of 100 μl media. Enriched cells were treated with 17AAG or NXD30001 at different concentrations, or then exposed to 3 Gy X-ray radiation for 1 min after 4 h. Proliferation assays *in vitro* testing were performed by counting the increase in viable cell numbers over 5 d using CellTiter-Glo Luminescent Cell Viability Assay (Promega, Madison, WI). After 30 min incubation at room temperature, the signal from the viable cells was analyzed on a Molecular Devices Spectramax M5 (Molecular Devices, California, USA). Replicate measurements were analyzed with respect to dose and estimates of half maximal inhibitory concentrations (IC50) were calculated by logistic regression (GraphPad Prism 5.0). Averaged cell titer of the sham-treated control group on day 1 was assigned a value of 1. All other relative cell titers were normalized accordingly.

To measure neurospheres formation, CD133+ glioma cells were seeded in 24-well plates at 100 cells per well. Spheriod cultures were grown for 1–2 d prior to increasing dose of NXD30001 incubation. Cells were allocated into 3 groups and treated with NXD30001 for 24 h, then irradiated for 1 min at 1, 2, or 3 Gy, each group has four replicates. At 7 d after plating, neurospheres containing more than 50 cells were scored.

To detect apoptosis, the cell number was measured daily for 3 d was using the CellTiter-Glo Luminescent Cell Viability Assay (Promega, Madison, WI), the caspase 3/7 activities at 72 h after NXD30001 alone or with 3 Gy radiation treatment were measured using the Caspase-Glo3/7 assay (Promega, Madison, WI) according to the manufacturer’s instructions. The caspase activities were normalized to the corresponding cell titers to generate the relative caspase 3/7 activities.

### Flow Cytometry

For apoptosis/necrosis detection, the CD133+ cells were grown in six-well plate (for suspension cells, Sarstedt) at a starting concentration of 5×10^4^ cells per well. Then CD133+ cells (10×10^5^ cell/ml) were treated of NXD30001 (2.5, 5, 10 nM) with or without radiation or vehicle (DMSO 0.1%) for 72 h. At the desired time point, 2×10^6^ cells were spun at 500 g for 5 min at 4°C and washed with PBS. Pellets were resuspended in 1 ml of cold PBS and added dropwise while gently vertexing to 9 ml 70% ethanol in a 15 ml polypropylene centrifuge tube. Fixed cells were then frozen at –20°C overnight. The next day, cells were centrifuged at 500 g for 10 min at 4°C and washed with 3 ml of cold PBS. Cells were resuspended in 500 μl of propidium iodide staining solution (0.2 mg/ml RNAse A, 0.02 mg/ml propidium iodide, 0.1 % Triton-X in PBS) and incubated for 20 min at 37°C. The cells were washed and resuspended in Annexin-V/propidium iodide buffer solution containing Annexin V-FITC (BD Pharmigen, 51-65874X) and Propidium Iodide (BD Pharmigen, 51-66211E). Samples were immediately analyzed on a five-laser BD LSRII. Visualizations and analyses of apoptotic fractions were generated using BD FACSDiva™ software.

### Immunoblotting Analysis

Lysates for blotting were prepared by seeding 5×10^5^ of the GBM cells onto culture dish, cells were treated with 17-AAG or NXD30001 at different concentrations with or without radiation at 3 Gy for various time points. After collecting the cells the pellets were washed three times with ice-cold PBS and the pellet after the final wash was resuspended in lysis buffer containing NP40, 50 mM Tris HCl (pH=8.0), 120 mM NaCl, 5 mM EDTA, 1% protease inhibitors (Roche Diagnostics, Indianapolis, IN), and phosphatase inhibitors (Roche Diagnostics, Indianapolis, IN). Protein concentrations were determined by using the Bio-Rad DC protein assay (Bio-rad, California, USA). Samples were loaded into a Novex Tris-Glycine Gel (Life Technologies, Grand Island, NY) and separated by electrophoreses at 125 V. The gels were then transferred onto a PVDF membrane (Immobilon-P; Millipore, Billerica, MA) by a wet Bio-Rad trans-blot system (Bio-rad, California, USA) and blocked by incubation with 5% dry milk in TBST (TBS with 0.1% Tween20). Primary antibodies were added to blocking solution and incubated overnight at 4°C on a shaker. Blots were washed several times with TBS-T-BSA. Chemiluminescent detection was performed with appropriate secondary antibodies. To quantify equal loading, the membranes were re-probed with a primary antibody targeting β-actin.

### *In Vivo* Assay With an Orthotopic Glioblastoma Model

All animal experiments were performed according to approved Institutional Animal Care and Use Committee (IACUC) protocols at Vanderbilt University Medical Center. T4302 CD133+ cells were implanted into the right cerebrum of female athymic nude mice for intracranial glioblastoma induction, as described previously (Cheng et al., 2013; Ma et al., 2015; Gong et al., 2016; Ma et al., 2017). Briefly, mice were anesthetized and placed in a small animal stereotactic frame (ASI Instruments, Houston, TX). The injection location was 1.5 mm anterior to the coronal suture, 2.5 mm to the right of the sagittal suture, and 3–3.5 mm below the skull. Approximately 5×10^3^ tumor cells suspended in 10 μl phosphate buffered saline were injected over 2 min using a 26–27 gauge syringe (approximately 3 mm deep), and the needle was left in position for 5 min and then withdrawn slowly. Upon withdrawal of the needle, the muscle and skin were closed with 5–0 silk sutures immediately. Before implantation, we first infected T4302 CD133+ cells with lentivirus directing expression of firefly luciferase and screened by 1 µg/ml puromycin for 3 d. After implantation, the mice were maintained for 10 d for tumor establishment and randomly divided into four treatment groups (n=10 per group) including vehicle, NXD30001, radiation, and NXD30001 combined with radiation. In NXD30001 and untreated groups, mice were treated intraperitoneally with either NXD30001 at 50 mg/kg every other day (q.o.d. IP) or a similar volume of vehicle control for 3 weeks. In radiotherapy alone or combined with NX30001 groups, animals received 3 Gy X-ray radiation in the whole brain once per day alone for 6 consecutive days or received same radiation plan one day after the second dose of NXD30001. Mice were weighted every time before drug administration. Tumor progression was monitored by bioluminescence imaging weekly from day 10 before NXD30001 or/and radiation treatment. Mice were imaged after injection of 150 mg/kg of D-luciferin (Promega, Madison, WI) using a Xenogen IVIS Spectrum (Caliper Life Sciences, Hopkinton, MA). Quantification was performed using Living Image software Caliper Life Sciences, Hopkinton, MA) with standardized rectangular regions of interests covering the mouse trunk and extremities. Animals were sacrificed upon the development of apparent symptoms, such as lethargy or hunched posture. The median survival was determined by the Kaplan-Meier method using the GraphPad Prism 5.0 software.

### Statistical Analysis

Values are reported as the mean ± the standard error. GraphPad Software 5.0 (GraphPad Software, Inc.) was used to determine statistical significance with either two-tailed Student’s t test or ANOVA as indicated. Significance testing of survival ratio was performed by log-rank analysis.

## Results

### NXD30001 Preferentially Kills GSCs

**Figure 1:**
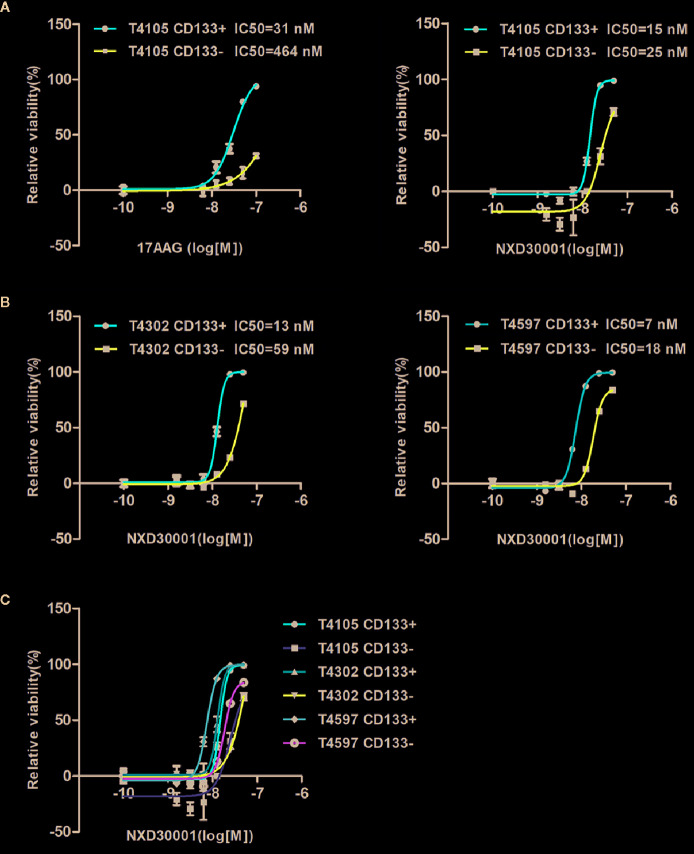
NXD30001 preferentially kills GSCs. **(A)** T4105 cells were treated with 17-AAG or NXD30001 at indicated concentrations by two-fold dilution. **(B)** Two pair cells of T4302 and T4597 were also used to detect the IC50 values for NXD30001. **(C)** The cell viability in CD133+ and CD133− cells including T4105, T4302, and T4597 were compared to find the discrepancy between them. IC50 values were determined and calculated by GraphPad Prism5. The cell viability was determined after 5-d incubation by CellTiter-Glo kits (Promega). The results are the average of triplicate samples. Error bars represent the standard error.

We detected the efficacy of 17-AAG and NXD30001 against GSCs, a clinically relevant model of human GBM as described before (Lee et al., 2006; Guerrero-Cazares et al., 2012). The results indicated that both 17-AAG and NXD30001 were more effective to T4105 CD133+ GBM cells in comparison to that in T4105 CD133− cells (**Figure 1A**). Same effect was also found in T4302 and T4597 pair cells with NXD30001 treatment (**Figure 1B**). **Figure 1C** showed that NXD30001 markedly decreased cell viability and proliferation in T4105, T4302, and T4597 GSCs cell lines. The growth of above CD133+ cell lines was markedly reduced by NXD30001 administration with IC50 values that ranged from 7 nM to 15 nM, while the IC50 values were higher in the three corresponding CD133− cells. Taken together, these data indicated that GBM CD133+ cells were more sensitive to NXD30001 than GBM CD133− cells.

### NXD30001 Promotes GSCs Sensitivity to Radiation

**Figure 2:**
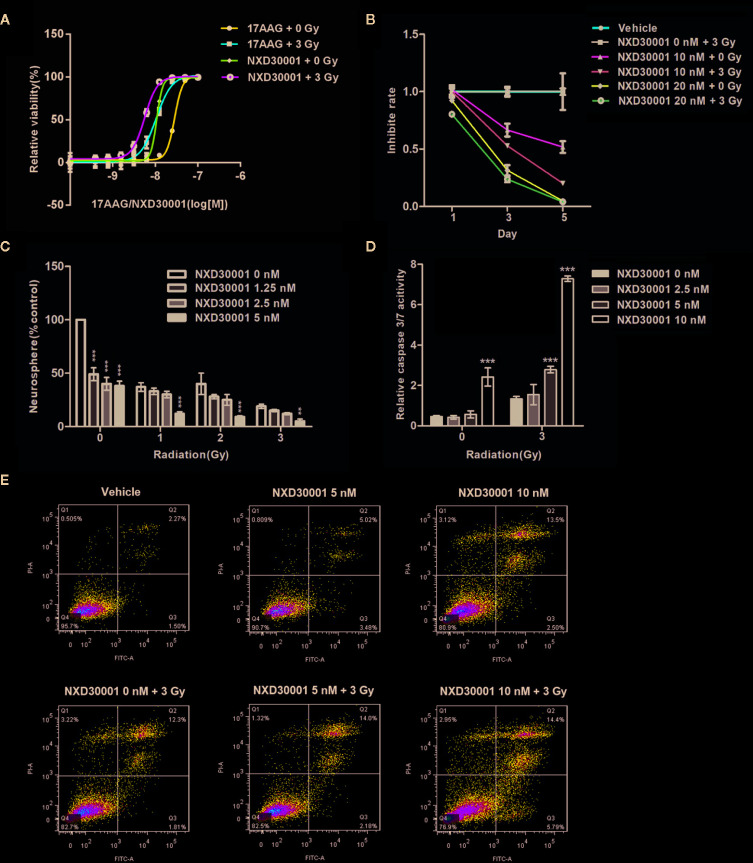
NXD30001 Renders GSCs More Sensitive to Radiation. **(A)** Cell viability assay of T4302 CD133+ cells treated with various concentrations of 17-AAG or NXD30001 with or without 3 Gy dose after 5-d incubation. **(B)** T4302 CD133+ cells growth was detected in the treatment with NXD30001 (10 and 20 nM) alone or combined with radiation after 5 d. **(C)** T4302 CD133+ cells were seeded at 100 cells per well in 24-well plate. Neurospheres were scored after 7-d incubation of NXD30001 without or with 1, 2, and 3 Gy of radiation. ^**^ *P* < 0.01, ^***^ *P* < 0.001. **(D)** The caspase 3/7 activity was determined 72 h after various treatments and normalized to cell titer of the corresponding treatment groups. The results are the average of triplicate samples. Error bars represent the standard error. ^**^ *P* < 0.01, ^***^ *P* < 0.001. **(E)** The Annexin V-FITC and flow cytometric assay was performed to detect apoptosis/necrosis. The dual parameter dot plots cells in the upper right quadrant were represent as late apoptosis (AnnexinV^+^-PI^−^), whereas cells in lower right quadrant were represent as early apoptosis (AnnexinV^+^-PI^+^).

In order to determine inhibitory potency of NXD30001 combination with radiation on GSCs, the neurosphere formation and Caspase-Glo3/7 assays were performed in T4302 CD133+ cells. The cell viability assay demonstrated that the radio-sensitivity of GSCs was more evident when treated with NXD30001 in comparison with 17-AAG (**Figure 2A**). More severely impaired cell growth with dose response was showed in treatment with NX30001 and 3 Gy radiation than NX30001 alone with days (**Figure 2B**). Neurosphere formation of GSCs was substantially reduced by NXD30001 with or without radiation in a concentration-dependent manner (**Figure 2C**), which indicated that NXD30001 might reduce the self-renewal potential of GSCs and lead to a reduction in tumor recurrence capacity. Caspase-Glo3/7 assay result revealed that the caspase activation of GSCs was significantly higher in 10 nM NXD30001 monotherapy group, and this effect was particularly prominent in treatment with NXD30001 + radiation (**Figure 2D**). Furthermore, the Annexin-V assay and flow cytometry confirmed that the number of Annexin-V and V/PI positive cells increased dose-dependently when treated with NXD30001 + radiation (**Figure 2E**), indicating that NXD30001 enhances apoptosis after radiation exposure. Above all, out study demonstrated that NXD30001 exhibited a radio-sensitization potential for GBM through inhibiting proliferation and inducing apoptosis of GSCs.

### NXD30001 Decreases Phosphorylation Levels of Multiple Hsp90 Client Proteins in Combination With Radiation in GSCs

**Figure 3:**
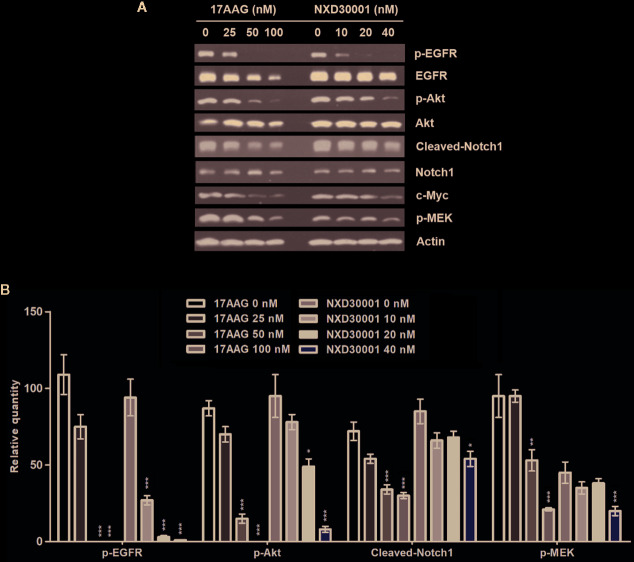
NXD30001 or 17-AAG dose-dependently decreased phosphorylation levels of multiple Hsp90 client proteins in GSCs. **(A)** Representative western blotting bands showing the expression levels of p-EGFR, EGFR, p-Akt, Akt, Cleaved-Notch1, Notch1, c-Myc, p-MEK in T4105 CD133+ cell cultures treated with different concentrations of 17-AAG or NXD30001. The result was quantified from the average of three different samples. **(B)** Quantification of western blotting bands. Error bars represent the standard error. ^*^ *P* < 0.05, ^**^ *P* < 0.01, ^***^ *P* < 0.001.

**Figure 4:**
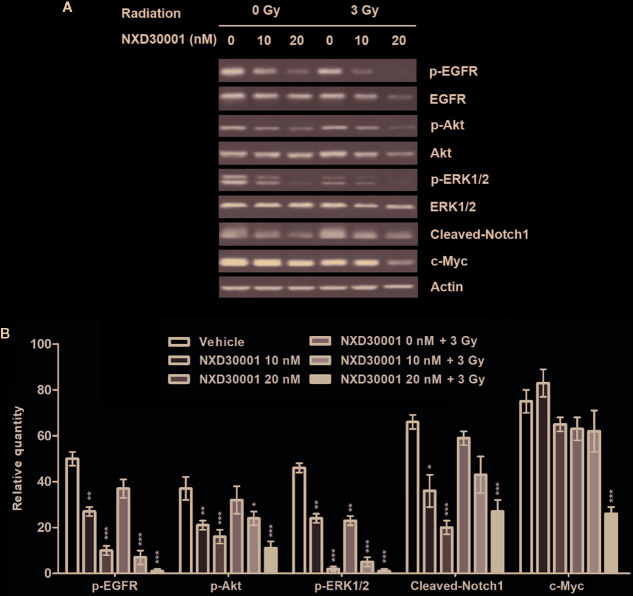
NXD30001 dose-dependently decreased phosphorylation levels of multiple Hsp90 client proteins in combination with radiation in GSCs. **(A)** Representative western blotting bands showing the expression level of p-EGFR, EGFR, p-Akt, Akt, p-ERK1/2, ERK1/2, Cleaved-Notch1, c-Myc in T4302 CD133+ cell cultures treated with different concentrations of NXD30001 with or without radiation. The result was quantified from the average of three different samples. **(B)** Quantification of western blotting bands. Error bars represent the standard error. ^*^ *P* < 0.05, ^**^ *P* < 0.01, ^***^ *P* < 0.001.

To determine the mechanism by which NXD30001 achieves its antitumor and radio-sensitizing effects on GSCs, we assessed the levels of known Hsp90 client proteins involved in GBM pathology when treated with 17-AAG or NXD30001 in T4105 CD133+ cells and treated with NXD30001 ± 3 Gy radiation in T4302 CD133+ cells. In T4105 CD133+ cells, 17-AAG and NXD30001 were shown to induce the degradation of Hsp90 client proteins, such as p-EGFR, p-Akt, c-Myc, Cleaved-Notch1, and p-MEK in a dose-dependent manner (**Figure 3**). The expressions of most client proteins began to decrease after 20 nM NXD30001 or 50 nM 17-AAG treatment, which indicated that NXD30001 had a stronger cell growth inhibiting effect than 17-AAG (**Figure 3**). In T4302 CD133+ cells, significant downregulation of Hsp90 client proteins, including p-EGFR, p-Akt, p-ERK1/2, and Cleaved-Notch1 was found when treated with NXD30001 alone, and this effect was more markedly after combined NXD30001 and radiation therapy (**Figure 4**). Although no significant variation of c-Myc protein expression was found in NXD30001 monotherapy group, c-Myc level decreased significantly when treated with 20 nM NXD30001 + radiation (**Figure 4**). These preliminary results suggested that NXD30001 may regulate radio-resistance pathways in GSCs through, at least in part, stabilization of important pro-survival factors, such as EGFR, Akt, Notch1, and c-Myc.

### NXD30001 Reduces Activation of DNA Damage Response in GSCs After Radiation

**Figure 5:**
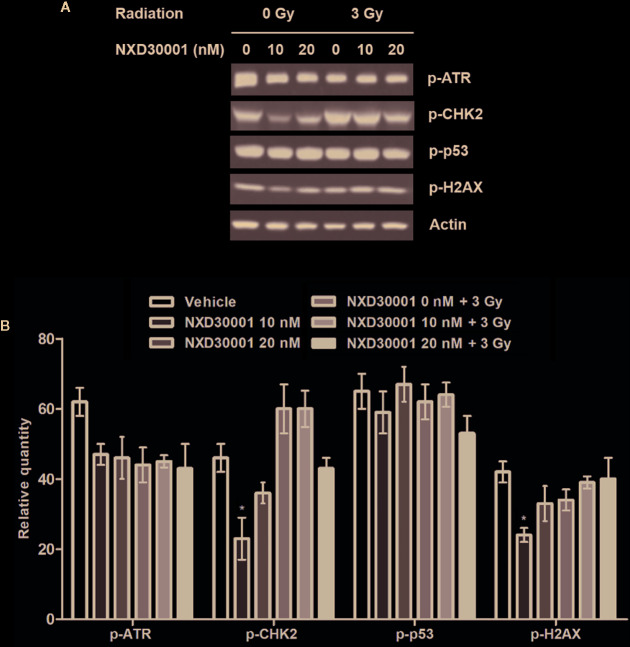
NXD30001 reduces activation of DNA damage response in GSCs after radiation. **(A)** Representative western blotting bands showing the expression level of p-ATR, p-CHK2, p-p53, p-H2AX in T4302 CD133+ cell cultures treated with different concentrations of NXD30001 with or without radiation. The result was quantified from the average of three different samples. **(B)** Quantification of western blotting bands. Error bars represent the standard error. ^*^ *P* < 0.05.

GSCs confers radio-resistance through preferential activation of the DNA damage checkpoint response to increase DNA repair capacity, which initiate tumor recurrent after radiotherapy (Bao et al., 2006; Yamamori et al., 2013; Skvortsova et al., 2015). The present data showed that the phosphorylation levels of DNA damage related proteins including ATR, CHK2, p53, and H2AX were not significantly altered in T4302 CD133+ cells following NXD30001 treatment with radiation, while significant down-regulation of p-CHK2 and p-H2AX levels was found in 10 nM NXD30001 monotherapy group (**Figure 5**). These results demonstrated that NXD30001 might reduce activation of postradiation DNA damage response in GSCs, and is speculated to impair survival signaling to induce GSCs death after radiotherapy.

### NXD30001 in Combination With Radiation Impairs ER Stress in GSCs

**Figure 6:**
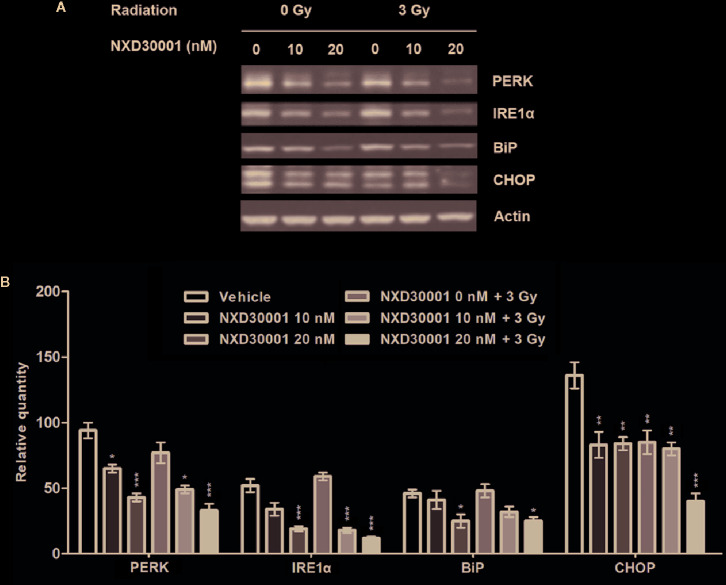
NXD30001 in combination with radiation impairs ER stress in GSCs. **(A)** Representative western blotting bands showing the expression level of PERK, IRE1α, BiP, CHOP in T4302 CD133+ cell cultures treated with different concentrations of NXD30001 with or without radiation. The result was quantified from the average of three different samples. **(B)** Quantification of western blotting bands. Error bars represent the standard error. ^*^ *P* < 0.05, ^**^ *P* < 0.01, ^***^ *P* < 0.001.

Unfolded protein response (UPR) that induced by ER stress response in cancer plays an important role in resistance to chemotherapy or radiation (Sui et al., 2013). In GSCs, the expression levels of UPR related proteins, such as PERK, IRE1α, GRP78/BiP, and CHOP were increased by activation of ER stress to keep cells survival (Yamamori et al., 2013; Skvortsova et al., 2015; Le Reste et al., 2016). To determine whether NXD30001 and radiation treatment result in impairing ER stress response to induce GSCs apoptosis, the expressions of the above ER stress sensors were detected in T4102 CD133+ cells. The results showed that PERK, IRE1α, BiP, and CHOP expression levels were significantly downregulated with NXD30001 administration, and the effect was more conspicuous when treated in combination with radiation (**Figure 6**). These data provide evidence regarding the suppressive role of NXD30001 ± radiation on GSCs through impairing ER stress.

### NXD30001 in Combination With Radiation Represses Orthotopic GBM

**Figure 7:**
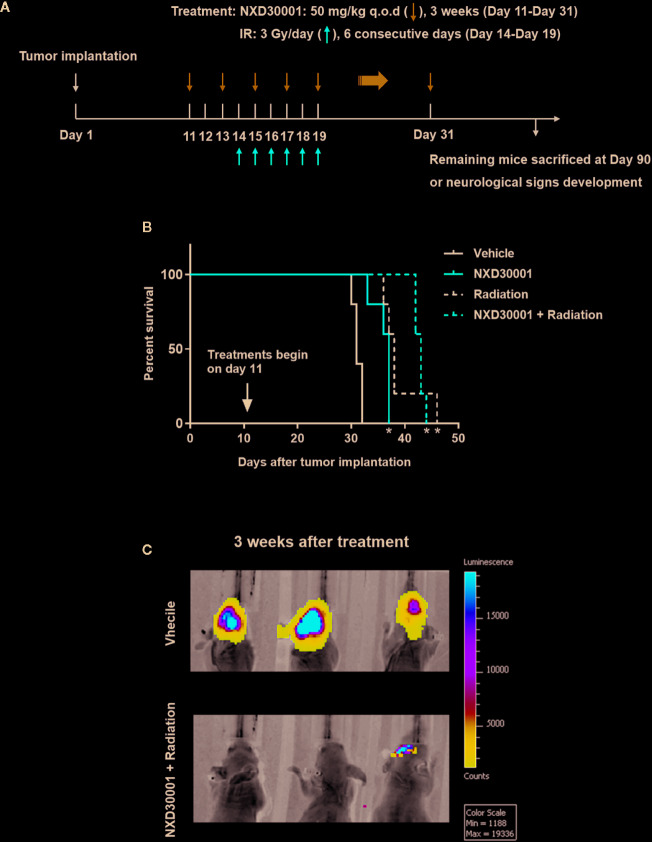
NXD30001 in combination with radiation represses orthotopic GBM. **(A)** T4302 GSCs was implanted into the right cortex of 4–6 weeks old athymic nude mice. Mice were maintained for 10 d to allow tumor establishment and randomized prior to treatment. From day 11, NXD30001 was administrated intraperitoneally at 50 mg/kg q.o.d. for 3 weeks. One day after the second dose of NXD30001, mice received radiation in the whole brain for 6 consecutive days at 3 Gy/day. The rest mice in each group were maintained upon neurological signs developed. **(B)** The survival percentage of tumor-bearing mice analyzed using Kaplan-Meyer curves. ^*^ *P* < 0.05 vs vehicle treated group. **(C)** A representative bioluminescence images of tumor-bearing mice 3 weeks after NXD30001+radiation treatment.

We evaluated the therapeutic potential of combination of NXD30001 and radiotherapy by using orthotopic GBM model *in vivo*. The administration of NXD30001 and irradiation plan was showed in **Figure 7A**. Without any treatment, mice implanted with GSCs were expected to die around 30 d according to our previous studies (Cheng et al., 2013; Ma et al., 2015; Gong et al., 2016; Ma et al., 2017). Treatment with NXD30001 alone extended the median survival time of tumor-bearing mice by approximately 20% of the vehicle subjects (37 d vs. 31 d, *P*<0.05, **Figure 7B**). Radiation alone increased the median survival of tumor-bearing mice from 31 d to 38 d (*P*<0.05), while radiation in combination with NXD30001 extended median survival to 43 d (*P*<0.05, **Figure 7B**). The bioluminescence imaging results have shown that the growth rate and volume of tumors in combined NXD30001 and radiation group were significantly lower than those in vehicle group (**Figure 7C**). These *in vivo* results were well concordant with that of *in vitro* assays, suggesting that NXD30001 in combination with radiation would be an effective treatment for glioblastoma.

## Discussion

Glioblastomas is one of the most lethal and frequent form of primary brain tumors, which undergoes continuous uncontrolled cell growth and proliferation. It is characterized by highly aggressive growth, invasiveness, and poor prognosis due to genetic and signaling abnormalities, which leads to an innate resistance to current therapies including radiation, alkylating chemotherapeutic agent temozolomide, and surgery (Gittleman et al., 2018). In this study, we assessed the antitumor effect of a brain-penetrating Hsp90 inhibitor, NXD30001 on GSCs *in vitro* and *in vivo* as a monotherapy or in combination with radiation. Our results showed that combined NXD30001 and radiotherapy may improve the antineoplastic activity in GBM through inhibiting GSCs proliferation and enhancing cell apoptosis by attenuating phosphorylation of multiple Hsp90 client proteins. NXD30001 also increased radio-sensitivity of GSCs through impairing DNA damage response and ER stress. Furthermore, NXD30001 in combination with radiation exerts better inhibitive effect on *in vivo* GBM growth, and provides a statistically significant prolonged survival of GBM-bearing mice.

CD133 accompanying with other neural and hematopoietic stem cell marker including Musashi-1, Nestin, Sox2, and Olig2 can identify glioma stem cells from different molecular subtypes of glioma. CD133+ GBM cells exhibit transcription profiles resembling the proneural subtype, whereas CD133− GBM cells may be associated with gliomas of the mesenchymal subtype (Joo et al., 2008; Lottaz et al., 2010). It has been reported that CD133+ glioma cells were sufficient to develop xenografted tumors *in vivo* that recapitulated the heterogeneity of the original tumor, and thus is considered as GSCs (Singh et al., 2004; Beier et al., 2007). GSCs has been shown more resistant to current therapeutic approaches than the matched non-stem glioma cells (Bao et al., 2006). Previous study has confirmed that GSCs was more sensitive to NXD30001 than non-GSCs (Zhu et al., 2010), which is inconsistent with our findings in this study.

Currently, the unsatisfactory therapeutic outcome in GBM are usually caused by the upregulation in expression of drug efflux transporters, reduced sensitivity to apoptotic signals, and increased expression of growth factors (Aldape et al., 2015). GBMs exhibit various signaling abnormalities, including EGFR amplifications, inactivating PTEN mutations, PDGF autocrine loops, and subsequent overactivation of their associated downstream signal generators. It is noteworthy that many of these proteins are Hsp90 client proteins and are responsible for initiation and maintenance of tumor (Pratt and Toft, 2003; Dutta Gupta et al., 2019). Thus, inhibiting Hsp90 activity will lead to downregulation of multiple oncogenic molecules and Hsp90 inhibitors may be effective chemotherapeutic agents for the treatment of GBM (Matts and Manjarrez, 2009). NXD30001 is a novel radicicol-based series of Hsp90 inhibitors and readily crosses the BBB and accumulates in the brain, made it be an attractive therapeutic candidate for GBM. Our study showed a significant inhibition of GSCs proliferation and induction of GSCs apoptosis after treatment with NXD30001 alone or combined with radiation, and these effects may be achieved through downregulating the phosphorylation levels of multiple Hsp90 client proteins including EGFR, EGFR, Akt, and c-Myc.

It is well known that radiotherapy could effectively repress GBM cell growth and induce cell death by leading to various types of DNA damage (Hathout and Pope, 2016). However, it is usually limited widespread used in curing GBM due to inherent tumor radiation resistance that caused by a DNA damage response (DDR) cascade, which is able to repair DNA damage and injury in surrounding normal tissues (Frosina, 2010; Schmalz et al., 2011; Alexander et al., 2012). When DNA damage initiates in tumor cells, the DDR is also activated to induce cell cycle arrest and lesion repair, which lead to tumor growth and recurrence. However, when the damage exceeds capacity of DNA repair, apoptosis will be triggered, further leading to tumor cell death. Generally, the activation of DDR is implicated in radio-resistance of the GSCs and it involved a range of DNA damages related proteins such as ataxia telangiectasia Rad3-related (ATR), which initiates a transduction cascade activating downstream effectors, including H2AX histone, 53 binding protein 1 (53BP1), and the checkpoint kinases Checkpoint 1 (CHK1) and Checkpoint 2 (CHK2) (Kastan and Bartek, 2004; Norollahi et al., 2019). It has been reported that there was an aberrant constitutive activation of DDR by upregulation of related proteins under DNA replication stress produced by oncogenes in GBM (Bartkova et al., 2010). In our study, the expression levels of DNA damage related proteins like ATR, CHK2, p53, and H2AX were not significantly altered in GSCs following NXD30001 treatment combined with radiation, indicating that NXD30001 was able to enhance the radio-sensitivity of GBM by reduction of the DNA damage response after radiotherapy.

ER stress is another important response involved in tumor development and growth. It is induced when the ER failed in maintaining cellular homeostasis, which is caused by constantly exposure to both intrinsic stresses like genomic instability, increased metabolic burden, oncogene expression, and extrinsic stresses, such as hypoxia, oxidative stress, and nutrient deprivation (Wang et al., 2010a). Cancer cells usually have stronger ability in protein synthesis and folding by inducing UPR through the activation of ER transmembrane protein sensors (Roy and Kumar, 2019). The UPR related proteins, such as protein kinaseR–like ER kinase (PERK), inositol-requiring enzyme 1a (IRE1α), binding immunoglobulin protein (BiP/GRP78) and transcription factor C/EBP homologous protein (CHOP) could be suppressed to mitigate ER stress condition for cancer survival including GBM (Le Reste et al., 2016; Penaranda Fajardo et al., 2016; Madden et al., 2019). In addition, ER stress response in cancer is reported to act as a key driver in tumorigenesis and the development of resistance to chemotherapy or radiation via activation of UPR (Bahar et al., 2019). Therefore, we speculate that reduction of ER stress response may impair cellular homeostasis and lead to cell apoptosis, which might be an effective strategy for GBM therapy. Our results showed that the expression levels of PERK, IRE1α, BiP, and CHOP proteins were decreased dose-dependently in the treatment of NXD30001 alone or combined with radiation in GSCs, suggesting that NXD30001 could improve radiation sensitivity through impairing ER stress and cell survival response ultimately leading to GSCs death.

Notably, although Hsp90 inhibitor NXD30001 has shown effective effects in enhancing radiosensitivity of GBM by targeting multiple radio-resistant pathways in our study, it is premature to conclude that the combination of NXD30001 and radiotherapy is a promising candidate to enhance GBM treatment. An effective animal model that better mimics the radiosensitivity of GBM is needed to be used in the future studies in order to irradiate with a dose closer to those used to treat GBM patients.

## Conclusion

The present study demonstrated that the Hsp90 inhibitor, NXD30001 could inhibit GSCs growth and proliferation and induce GSCs apoptosis as a monotherapy. Moreover, NXD30001 markedly increased radio-sensitivity of GSCs through decreasing phosphorylation levels of multiple Hsp90 client proteins, and impairing DNA damage response and ER stress response. In an orthotopic GBM model, NXD30001 in combination with radiotherapy could significantly inhibit tumor growth and extend median survival of tumor-bearing mice, which provides a valuable basis for its use in the treatment of GBM.

## Data Availability Statement

The original contributions presented in the study are included in the article/supplementary material; further inquiries can be directed to the corresponding authors.

## Ethics Statement

The animal study was reviewed and approved by Vanderbilt Institutional Animal Care and Use Committee.

## Author Contributions 

HC, ZC, and LX conceived and designed the experiments. HC and ZC performed the experiments and analyzed the data. YG and YM contributed reagents, materials, and analysis tools. RT and JW searched and reviewed the literatures. HC drafted the paper. ZC and LX critically revised the article. All authors contributed to the article and approved the submitted version.

## Funding

HC was partly supported by Shanghai Pujiang Program (18PJD034). LX was partly supported by National Natural Science Foundation of China (81801169). ZC was partly supported by grants from the National Natural Science Foundation of China (81372395). JW was supported by the American Brain Tumor Association and the Southeastern Brain Tumor Foundation NIH/NCI grant (1R01CA166492).

## Conflict of Interest

The authors declare that the research was conducted in the absence of any commercial or financial relationships that could be construed as a potential conflict of interest.
